# Risk Analysis of Cellulose Nanomaterials by Inhalation: Current State of Science

**DOI:** 10.3390/nano9030337

**Published:** 2019-03-02

**Authors:** James D. Ede, Kimberly J. Ong, Michael Goergen, Alan Rudie, Cassidy A. Pomeroy-Carter, Jo Anne Shatkin

**Affiliations:** 1 Vireo Advisors, LLC, Boston, MA 02130-4323, USA; jede@vireoadvisors.com (J.D.E.); kong@vireoadvisors.com (K.J.O.); cpomeroy@vireoadvisors.com (C.A.P.-C.); 2 P3Nano, U.S. Endowment for Forestry and Communities, Greenville, SC 29601, USA; michael@usendowment.org; 3 Forest Products Laboratory, USDA Forest Service, Madison, WI 53726-2398, USA; arudie@fs.fed.us

**Keywords:** cellulose, nanomaterial, inhalation, risk assessment, safety, nanocellulose, review

## Abstract

Cellulose nanomaterials (CNs) are emerging advanced materials with many unique properties and growing commercial significance. A life-cycle risk assessment and environmental health and safety roadmap identified potential risks from inhalation of powdered CNs in the workplace as a key gap in our understanding of safety and recommended addressing this data gap to advance the safe and successful commercialization of these materials. Here, we (i) summarize the currently available published literature for its contribution to our current understanding of CN inhalation hazard and (ii) evaluate the quality of the studies for risk assessment purposes using published study evaluation tools for nanomaterials to assess the weight of evidence provided. Our analysis found that the quality of the available studies is generally inadequate for risk assessment purposes but is improving over time. There have been some advances in knowledge about the effects of short-term inhalation exposures of CN. The most recent in vivo studies suggest that short-term exposure to CNs results in transient inflammation, similarly to other poorly soluble, low toxicity dusts such as conventional cellulose, but is markedly different from fibers with known toxicity such as certain types of multiwalled carbon nanotubes or asbestos. However, several data gaps remain, and there is still a lack of understanding of the effects from long-term, low-dose exposures that represent realistic workplace conditions, essential for a quantitative assessment of potential health risk. Therefore, taking precautions when handling dry forms of CNs to avoid dust inhalation exposure is warranted.

## 1. Introduction

Cellulose nanomaterials (CNs) are emerging materials with numerous applications. They have the potential to be used in high volumes for cement, automotive composites, food packaging, paper and coatings, consumer product packaging, hygiene and absorbent products, and as textiles for clothing, among many other applications [1]. To promote the safe commercialization of these materials, a life-cycle risk assessment and environmental health and safety roadmap identified key knowledge gaps in our understanding of CN safety and prioritized them for development [2]. The assessment found that improving understanding of the risks of inhaling dry CN powders in the workplace is a high priority.

As the commercialization of CNs continues to grow, inhalation of particles into the lung is one of the main routes of exposure, especially, in occupational settings where workers may be exposed to concentrated doses of airborne, dry particulate materials. It is well-understood that inhalation of poorly soluble, low toxicity (PSLT) dusts, such as silica, titanium dioxide, and coal mine dusts, has the potential to irritate the lungs [3] and trigger the immune system; effects may occur when individuals are exposed to either short-term to high levels of PSLT dust, or long-term at low levels that exceed the lowest adverse effect threshold. CNs are bio-based, inert materials that may be similar in risk *potential* to PSLT dusts. However, due to their smaller size and fibrillar form, CNs should be assessed for their potential to be respirable and for their inflammatory effects that may lead to serious health outcomes, such as carcinogenicity. The main forms of wood-based CNs are cellulose nanocrystals (CNCs) and cellulose nanofibrils (CNFs). Both forms are extracted from plants via a purification and homogenization pre-treatment step, followed either by an acid hydrolysis refinement to produce CNCs or mechanical shear to produce CNF. CNCs are smaller and tend to be stiff, with lengths between 50–350 nm and widths of 5–20 nm, while CNFs are flexible, with lengths typically >1 µm and widths of 20–100 nm [4]. In this study, we evaluated the available literature to assess whether, due to their properties, CNs behave differently to conventional cellulose dust, a known respiratory irritant, and, therefore, require different occupational handling approaches.

Performing well-designed inhalation studies for risk assessment purposes is difficult, and testing nanomaterials comes with additional challenges. Considerations for delivery method, dose selection, control groups, and study duration are crucial to identifying outcomes relevant for risk assessment. Repeat, low-level exposures may not be well represented by short-term high-dose studies, but long-term studies require time and extensive resources. A number of groups have highlighted the need for quality assessment of nanotoxicology studies [5,6,7]. Toxicity studies of nanomaterials require material characterization and special experimental design considerations for the results to meaningfully contribute to an understanding of safety. Recent reviews highlight that a significant number of studies being published on nanomaterials do not meet these criteria, limiting their usefulness for risk assessment [8,9]. This review evaluates the current state of knowledge in relation to the quality of CN inhalation studies for risk assessment purposes using two published approaches: Krug and Wick (2011) [10] and Card and Magnuson (2010) [11]. The criteria do not indicate whether a study is “good” or “bad” but rather if the study was designed and conducted to allow findings to be used for risk assessment purposes to help predict negative biological outcomes as a result of CN inhalation exposure. There is a bias in published toxicity studies, which tend to report on short-term exposures at concentrations that result in negative biological effects and focus on the mechanistic aspects of toxicology, such as determining the mode and mechanism of action. A key challenge for risk assessment is extrapolating the information to assess how these same materials may behave under realistic scenarios, where concentrations are often significantly lower and exposures can be repetitive or prolonged. This analysis focuses on: (1) Reviewing the quality of the studies for risk assessment purposes using published study evaluation tools for nanomaterials to assess their impact on the weight of evidence, and (2) reviewing the results of these papers for their contribution to the current understanding of occupational inhalation hazards of dried CN in the workplace.

## 2. Literature Review

To date, twelve studies from 2011–2018 have been published on the short-term inhalation toxicity of CNs [12,13,14,15,16,17,18,19,20,21,22,23]. These studies use both cellular (in vitro) [16,17,18,19,20,21] and animal (in vivo) [12,13,14,15,16,22,23] models to investigate the effect of CNC and CNF exposure on the lung. The main findings from these studies are summarized here before analyzing the studies using two published approaches for their usefulness for risk assessment purposes.

### 2.1. Cellular (in vitro) Studies

Acute inhalation toxicity of CN has been investigated in vitro using both 3D triple cell co-culture cell models of human airways and simpler monocultures. Initial investigations using the 3D barrier model examined incubation with aqueous suspensions of CNC (5, 15 and 30 mg/L for 24 h) and found apical cytotoxicity at concentrations of 15 and 30 mg/L, but no basolateral cytotoxicity at any dose examined, and only a small elevation of pro-inflammatory chemokine at the highest dose examined [17]. A more recent study by Endes et al. (2014) exposed the 3D human airway barrier model to aerosolized CNC suspensions [18]. The authors tested nebulized concentrations of CNC from 0.14 to 1.57 μg/cm^2^ and found no significant cytotoxicity, no induction of oxidative stress, and no pro-inflammatory response at any of the concentrations examined, 24 h post-exposure.

In a study using monocultures, Yanamala et al. (2016) exposed aqueous suspensions of different forms of CNC and CNF (5 μg/mL–300 μg/mL) to a human lung epithelial cell line (A549) or a human monocytic (immune) cell line (THP-1) for 24 or 72 h [19]. Bulk microcrystalline cellulose (MCC) was used as a reference material. The authors found responses were cell-type- and material-specific and concluded there was no correlation between cytotoxicity and surface properties. The authors also found elevated pro-inflammatory responses following CN exposure (50 μg/mL; 24 and 72 h) in THP-1 cells, with different responses observed between materials. The authors concluded that at the doses and time points examined, all forms of CNs were nontoxic or less toxic compared to the two positive controls used in the study: asbestos or lipopolysaccharide.

A similar study published by Menas et al. (2017) examined cytotoxicity, oxidative stress, and cytokine secretion in A549 cells following exposure (1.5, 15 or 45 μg/cm^2^) to various forms of CNC or CNF for 24 or 72 h [20]. Chitin and carbon nanofibers were used as reference materials. Generally, cell viability was significantly decreased at all doses examined following CNF, but not CNC, exposure for 72 h. CNC and CNF exposure generally resulted in significant oxidative stress responses at both time points; although, some differences were noted between materials. The authors also found that exposure to 45 μg/cm^2^ CNC for 72 h significantly increased the secretion of several pro-inflammatory cytokines in A549 cells.

Two more recent studies have further investigated cell responses to CNF exposure. Lopes et al. (2017) examined cytotoxicity, oxidative stress, and cytokine secretion following exposure to different surface-functionalized CNFs in human dermal, lung, and immune cells (human dermal fibroblasts, lung MRC-5 fibroblast and THP-1 monocytes), using MCC as a reference material [21]. The authors found that CNF exposure did not induce cytotoxicity in any of the cell lines examined up to 500 μg/mL for 24 h of exposure. The authors also found CNF exposure up to 500 μg/mL: (1) did not induce oxidative stress; and (2) did not result in internalization or morphological changes in THP-1 monocytes. Two pro-inflammatory cytokines were elevated in THP-1 cells exposed to unmodified CNF for 24 h starting at concentrations of 250 μg/mL; these effects were not observed for modified-CNF or the MCC reference material.

A similarly designed study by Ilves et al. (2018) examined the cytotoxicity and pro-inflammatory cytokine production in THP-1 cells following exposure to four surface-modified CNFs and compared these responses to conventional cellulose [16]. The authors found that one of the unmodified CNFs reduced cell viability and triggered pro-inflammatory cytokine production; the remaining three CNF materials had no significant changes and were similar to the response observed for bulk-sized cellulose. Cytotoxic effects of the unmodified CNF were observed after 3, 6, and 24 h of exposure, starting at concentrations of 10 μg/mL. Similarly, increased expression and protein production of pro-inflammatory cytokines were observed after 3, 6, and 24 h of exposure, starting at concentrations of 10 μg/mL.

### 2.2. Animal (In Vivo) Studies

Acute inhalation studies of CN have been conducted with both mice and rats. O’Connor et al. (2014) exposed rats to aerosolized CNC for four hours and monitored the animals for 14 days [22]. Researchers were only able to achieve a maximum test concentration of 0.26 mg/L and found no mortality, gross toxicity, adverse effects, or behavioral changes at the highest concentration tested. Yanamala et al. (2014) examined the effect of pharyngeal aspiration of two forms of CNC in C57BL/6 mice [12]. Mice were exposed to 100 or 200 μg CNC for four hours and monitored for 24 h. Analysis of bronchial alveolar lavage (BAL) fluid following CNC exposure found pulmonary damage, elevated cytokine and chemokine levels, and recruitment of inflammatory cells.

Two additional studies examined potential sex differences and reproductive effects in mice following pharyngeal aspiration to suspensions of CNC (40 µg/mouse/day; two times per week, for three weeks; cumulative dose of 240 μg/mouse) and observed responses three months post-exposure. Shvedova et al. (2016) reported pulmonary damage and an elevated oxidative stress response in BAL from exposed mice [14]. The authors also reported impaired pulmonary function and global changes in gene expression following CNC exposure. For many of these endpoints, the authors conclude that effects were markedly more pronounced in female compared to male mice, suggesting sex differences in response to CNC exposure, though these results may also be due to weight differences between sexes [24]. In a second study, Farcas et al., (2016) used the same mice to examine potential male reproductive effects following CNC exposure [13]. The authors found significant changes to sperm three months post-exposure. Evaluation of the testes found elevated oxidative stress, inflammatory cytokines, and myeloperoxidase (MPO) activity, and histopathological analysis found damage to testicular structure. Significant changes in hormone levels were also reported.

Two recent studies examined the acute inhalation toxicity of CNF in mice. Catalán et al. (2017) exposed mice to a one-time exposure of CNF via pharyngeal aspiration (10, 40, 80, or 200 μg/mouse) and found an acute inflammatory response and DNA damage 24 h post-exposure [15]. Histological analysis of lung tissue confirmed deposition and accumulation of CNF in the bronchi and alveoli, as well as internalization in macrophages. The authors also found DNA damage in isolated lung cells, though no dose–response relationship was observed. No DNA damage was found in cells isolated from BAL, and no chromosome damage was found in bone marrow erythrocytes.

Park et al. (2018) compared the pulmonary effects of four materials—CNF, CNC, single-walled carbon nanotubes (SWCNTs), and crocidolite asbestos. BALB/c mice were exposed to a single dose of 40 µg/mouse of SWCNT or asbestos, and 40 µg or 80 µg of CNF or CNC by pharyngeal aspiration and evaluated for pulmonary inflammation and immune response 14 days post-exposure. By day 14, all mice showed some cellular alterations indicative of an inflammatory response, such as increased total cell count, mononuclear phagocytes, and polymorphonuclear leukocytes, and lymphocytes in the BAL, increased expression of cytokines and chemokines, and increased lactic acid dehydrogenase (LDH) activity. CNF and CNC responses were generally dose-dependent, with higher doses activating a greater response. However, the immune response induced by asbestos was indicative of chronic inflammation, whereas the SWCNT, CNF, and CNC induced much weaker responses, dissimilar to asbestos, the positive control.

Ilves et al. (2018) recently published a study of one-time exposures using pharyngeal aspiration (10 or 40 μg/mouse) to one of four surface-modified CNFs in mice [16]. The authors included two observation timepoints (24 h and 28 days post-exposure), used positive and negative controls, and compared results to relevant reference materials—multiwalled carbon nanotubes (MWCNTs) and bulk-size cellulose. Twenty-four hours post-CNF exposure, the authors reported recruitment of inflammatory cells in BAL, with similar responses observed for both MWCNT and bulk cellulose exposures; however, differences between CNF materials were noted. CNF exposure enhanced mRNA expression of several pro-inflammatory cytokines 24 h post-exposure, though differences between CNF materials were observed; generally, similar cytokine responses were observed for both bulk-cellulose and MWCNT reference materials. Importantly, only modest immune reactions were observed 28 days post-exposure, with effects reduced compared to 24 h post-exposure for CNF and similar to those triggered by bulk cellulose. In comparison to MWCNTs, the effects of CNF 28 days post-exposure were more minor. The authors also reported that the CNs persisted in the lung 28 days post-exposure.

## 3. Study Evaluations—Krug and Wick and Card and Magnuson 

Two sets of criteria were used to evaluate the studies: One set developed by authors Krug and Wick (2011) [10] and another by Card and Magnuson (2010) [11]. Both sets of criteria were originally developed to assess the quality of nanotoxicity studies, recognizing that various studies have different objectives. Researchers may aim to determine the mechanisms of toxicity (i.e., how are these materials causing their effects at high doses), find the lowest observed adverse effect level (i.e., at what concentration do effects start to occur) or conduct a risk assessment, which considers whether a hazard might cause harm to exposed persons under realistic exposure scenarios. Here, we have adapted the Krug and Wick and Card and Magnuson criteria sets to specifically assess the quality of studies examining short-term CN inhalation for risk assessment purposes and used them to evaluate seven animal (in vivo) studies [12,13,14,15,16,22,23] and six cellular (in vitro) studies [16,17,18,19,20,21] (Table 1). Studies were evaluated that specifically examined exposure to CN using in vivo or in vitro models of inhalation; studies examining inhalation exposure to bulk cellulose were not included, except when included in the study design as reference materials.

### 3.1. The Krug and Wick Approach

For the past decade, Krug and Wick (2011) [10] have been working toward enhancing the quality and reliability of nanotoxicity studies. They stipulate that sufficient characterization and relevant information on the validity and suitability of the selected test methods should be required for nanotoxicology publications to ensure comparable studies, leading to reliable discussion and the ability to make a conclusive evaluation of the risks associated with exposure to certain nanomaterials.

Krug and Wick developed a set of criteria (Table 2) to evaluate nanomaterial toxicity studies based on (i) the extent of physical and chemical characterization and (ii) the overall study design (e.g., doses administered, exposure route) [10]. For our analysis, we quantified these criteria by assigning a category of 2 if fully met, 1 if partially met, and 0 if not met. Scores were then calculated for a final ‘nanomaterial characterization score’, out of 14, and a ‘study design score’, out of 20 (or 18, as one criterion only applies to in vivo studies). The criteria were adapted for evaluating inhalation studies with CN; therefore, the criteria for the octanol-water partition coefficient, solubility, and criteria for ecotoxicological studies were deemed not applicable and excluded.

### 3.2. The Card and Magnuson Approach

Card and Magnuson (2010) proposed a quantitative two-step method to assess nanotoxicity studies for quality [11]. First, a ‘study design score’ is calculated that assesses the adequacy and documentation of study design, methods, materials, and results using the Toxicology Data Reliability Assessment Tool (ToxRTool). For our analysis, the study design was assessed from a risk assessment perspective. The criterion, “Is the study design chosen appropriate for obtaining the substance-specific data aimed at?” was evaluated according to risk assessment principles, including appropriate exposure delivery, realistic dose and duration of exposure, evaluation of a dose–response relationship, and the inclusion of control groups [24]. Based on the criteria groups in the ToxRTool, the study design is scored as a: 1 (reliable study without restrictions), 2 (reliable study with restrictions), or 3 (unreliable study). In step 2, a ‘nanomaterial characterization score’ is calculated, based on the completeness of the physical and chemical characterization of the nanomaterial (Table 3). A score of 0 indicates limited or no characterization was completed; a score of 10 indicates thorough characterization. The results are graph-based on the score in both of these categories to depict the overall quality of the study for risk assessment. The authors envisioned that this approach could be used as a standardized method to assess manuscript quality or for regulatory review of nanotoxicity studies. Our calculation of the study design score specifically evaluated study design and reporting as it relates to risk assessment of dry CN powder via inhalation. As with the Krug and Wick approach, the user bears responsibility for critically evaluating and determining the relevance of the information it provides.

## 4. Study Evaluation—General Observations

The physical-chemical characterization criteria are similar between the two approaches, but the Card and Magnuson approach breaks down the criteria into more distinct categories, where chemical composition and purity, as well as shape and crystallinity, are each separate criteria for assigning a nanomaterial characterization score. Evaluating the CN studies using the criteria, we found that, in general, there was inadequate physical and chemical characterization of CNs, limiting the comparisons that can be made between studies and to other materials. However, over time, studies are improving and reporting more detailed physical and chemical characterization (Table 2 and Table 3).

**Figure 1:**
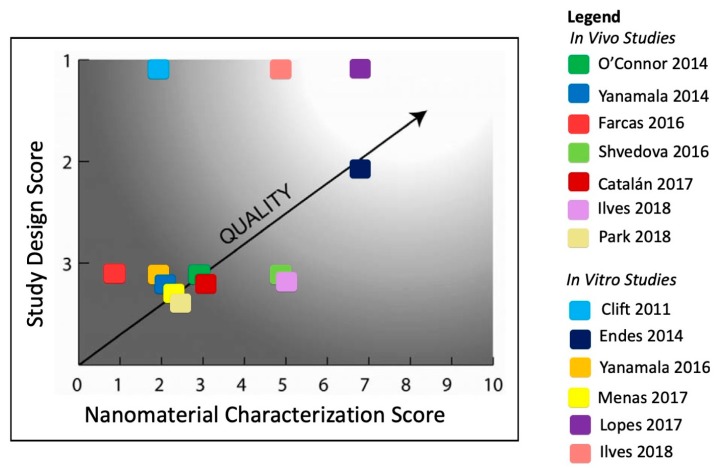
A schematic assessment of the overall quality of CN inhalation studies for risk assessment purposes based on its study design score and nanomaterial characterization score. The clear area represents a range of scores for which a study can be considered of high overall quality; conversely, the shaded area represents studies of low overall quality (adapted from Card and Magnuson, 2010 [11]).

Animal study design, as evaluated by both sets of criteria, reveals several weaknesses that reduce the value of the studies for risk assessment. In the Card and Magnuson approach, no animal studies received a high total score, as shown in Figure 1. Most studies received a study design score of 3, indicating significant deficiencies in study design, methods, materials, and/or reporting of results for risk assessment. This was largely the result of ‘mandatory minimum criteria’ in the ToxRTool used to assign study scores under the Card and Magnuson approach. If one of these mandatory minimum criteria is not met, the study is automatically assigned the lowest study design score of 3. For this assessment, none of the animal studies were able to meet this mandatory minimum criterion: “Is the study design chosen appropriate for obtaining the data aimed at?” For the purpose of this evaluation, this criterion was evaluated for obtaining data relevant for risk assessment purposes, which includes exposure delivery, dose and duration of exposure, evaluation of a dose–response relationship, and inclusion of control groups [24]. The study design scores under the Card and Magnuson approach were generally lower compared to the scores assigned under the Krug and Wick approach due to ‘mandatory minimum criteria’ and subsequent categorization to the lowest score of 3.

Unlike Card and Magnuson, which categorizes study design scores into groups 1, 2, or 3; the Krug and Wick approach is based on ten criteria for evaluating study design with an overall ‘study design score’ out of 20 for animal studies. Study design scores were generally low, ranging from 1–11. Most of the studies did not adhere to some of the basic rules in the design of dose–response toxicity studies, or were not designed for that purpose. In general, studies failed to: (i) Establish a dose–response curve; (ii) ensure that the doses being administered were realistic toward human exposures; and (iii) clearly identify “overload” conditions, where doses might cause overt toxicity and be unreliable for studying toxic effects. However, similar to nanomaterial characterization, over time, studies are improving their overall design and data reporting (Table 2 and Table 3).

## 5. Physical-Chemical Measurement and Reporting

Both the Krug and Wick and the Card and Magnuson approaches recognized that there is much uncertainty regarding the physical-chemical characterization necessary for a toxicology study. Some parameters may be more important for one nanomaterial or exposure scenario than they are for another; therefore, standardizing a list and weighting or ranking parameters was not possible. While an understanding of important parameters and the reliability of measurement has improved in nanomaterial studies since the list was developed, there are still few prescribed physical-chemical parameter lists, though some have been suggested (e.g., ECHA 2016 [25], Arts et al. 2015 [26], Oomen et al. 2015 [27]). Definitively linking physical-chemical parameters to biological activity is situation-specific. Attempts to group materials are ongoing [28], which guide the development of such nanomaterial- and situation-specific lists, though such recommendations are slow to be adopted [8].

Similar types of physical-chemical information are generally being provided in both in vitro and in vivo studies (Table 2,3). The Card and Magnuson evaluation shows that particle size/size distribution and shape is evaluated by most studies (100% and 92%, respectively). Surface charge (46%), chemical composition (31%), and purity (31%) were all measured by some studies, while agglomeration/aggregation (15%), crystallinity (8%), surface area (8%), surface chemistry (8%), and characterization in relevant media (15%) were only reported in one or two of the reviewed studies. Size and size distribution are standard reporting metrics for nanomaterial studies and are often measured with electron microscopy or atomic force microscopy [4]. Surface charge (typically measured as zeta-potential) may be an important metric to measure for insoluble nanomaterials, as the charge may affect the interaction of the nanomaterials with proteins and membranes, which could alter the risk of inflammation and lung injury [29,30]. The chemical composition and surface chemistry of CNs vary—for example, CNCs often have sulfate groups, whereas CNFs may not, affecting a myriad of physical-chemical properties [4]. Purity is considered a priority measurement for inhalation studies to distinguish test material from endotoxin or contaminant effects, as endotoxins are often a major inflammatory agent in dust [31,32]. Purity measurements may be particularly important for CNs, as they are derived from wood and plant sources that naturally contain microbes, and contamination with microbes or metals may also occur during manufacture and processing [4]. Agglomeration and aggregation properties modify available surface area and affect the uptake, translocation, and clearance in the lung [33]. Crystallinity is an important measurement for nanomaterials that have different crystal structures (e.g., rutile and anatase titanium dioxide) related to catalytic properties [34]; although, there are no indications that this is relevant for CNs. However, the degree of crystallinity is related to the stiffness of the materials. Stiffness is an important measure for fibrous materials, in which fibers of certain stiffness and length (such as glass and asbestos) can lead to chronic inflammation and eventually to more serious outcomes [35]. The relationship between surface area and pulmonary effects appears to be relevant for some nanomaterials but not others (e.g., Schmid and Stoeger 2016 [36], Warheit et al. 2006 [37]), so reporting these data will help distinguish whether there are any ‘nano-specific’ effects, or if an effect can be more straightforwardly attributed to scaling of the surface-area-to-volume ratio. The final criterion, characterization in relevant media, should be an essential component for all physical-chemical measurements. Most of the studies measured physical-chemical properties of their CNs in water, rather than the dispersion media or biologically relevant fluids; therefore, these data may not be representative of the nanomaterial in the exposure media, and in the lung.

A lack of physical-chemical characterization hinders the ability to use these data for risk assessment and to confidently apply the findings of these studies to other materials. The challenge of finding appropriate test methods, as well as an absence of standardized criteria, contributes to the generally poor scores for physical-chemical criteria. Some researchers may not publish their characterization data because they acknowledge that some measurements may not be reliable; for example, dynamic light scattering (DLS) has become the standard method to measure hydrodynamic size, or agglomeration and aggregation state. However, this method is designed for spherical particles; for CNs, which tend to be rod-like or fibrillar, DLS likely does not provide accurate readings, though in some cases DLS can be useful for measuring aggregation or colloidal stability [4]. Characterization methods and best practices continue to be developed, with most based on existing methods modified for CNs [4].

## 6. Study Design Considerations

### 6.1. In vitro versus in vivo Studies

In vitro studies tend to score higher on study design than in vivo methods (Table 2), with the exception of ‘at least two different tests for each biological end point’, which is at least partially met in every in vivo study under the Krug and Wick evaluation [10]. In addition, in vitro studies more often report the dose in more than one unit; concentration (µg/mL) and as the deposited dose per cell (µg/cell or µg/cm^2^), whereas in vivo studies tend to only report the dose as mass per mouse (µg/mouse). Over time, animal study designs have improved. Studies conducted from 2014–2016 received study design scores of 1–3, while the most recent studies in 2017 and 2018 received scores from 7–11 under the Krug and Wick approach [10]. This is attributed to improvements, including the use of positive, negative, and vehicle controls (including reference materials such as conventional cellulose and carbon nanotubes) [16] and longer study times more appropriate for assessing effects beyond a subacute period.

### 6.2. Exposure Technique

Most of the animal studies used a technique called pharyngeal aspiration to deliver high bolus (i.e., all at once) doses of CN to mouse lungs. This method does not accurately mimic the inhalation of CN that would occur in an occupational setting because it occurs in a moment rather than over time [24]. High-dose delivery of PSLT nanoparticles may elicit different inflammatory responses compared to low-dose delivery [38]. Even when standardized for similar lung loads, bolus delivery methods, such as intratracheal instillation, elicit an elevated inflammatory response compared to inhalation exposure [39]. High-dose bolus delivery studies may be useful for hazard ranking of materials, but these types of studies do not allow estimates of no- or lowest observed effect levels, from which to establish exposure limit values, and, therefore, are of limited utility for risk characterization [24]. Inhalation exposure is the gold standard and part of the Organisation for Economic Cooperation and Development (OECD) inhalation testing methods (e.g., OECD 412 and 413 for subacute and subchronic exposures). In inhalation studies, a test substance is aerosolized in a chamber, and the animal breathes in the substance in a more natural manner. The OECD does endorse the use of pharyngeal aspiration as a simple and inexpensive method to rapidly screen and rank substances, including fibers and nanomaterials [40] but recommends that results be supported by an inhalation study [41].

### 6.3. Exposure Dose and Duration

Most of the studies examined only high doses of CNs that are not representative of realistic levels of exposure. A previous assessment of one of these papers suggests that simulating these high doses by inhalation would require unrealistic workplace exposure concentrations in the g/m^3^ range [24]. The U.S. National Institute for Occupational Safety and Health (NIOSH) has conducted exposure assessments in several U.S. governmental, academic, and industrial pilot production facilities and generally found very low total exposure levels, well below 100 µg/m^3^ [42]. After assessment of four production facilities, the maximum estimated concentration of detected airborne cellulose was more than ten times below the U.S. Occupational Safety and Health Administration’s 5 mg/m^3^ permissible exposure limit for the respirable fraction of cellulose dust, which occurred during the milling and cutting of a CNC polymer composite (NIOSH Nanotechnology Field Studies team, personal communication, 19 June 2018). Measurement of airborne CN in full-scale commercial manufacturing facilities will be critical to establish realistic exposures prior to commencing resource-intensive long-term inhalation studies.

Instillation or aspiration of excessive doses, or exposure to high dose-rates of nanomaterials, may overwhelm the integrity of the pulmonary surfactant and permanently compromise lung mechanics [43]. As such, we do not know whether the effects observed in the studies might occur at more likely exposure levels which would be at much lower concentrations over longer periods of time. Further, many studies observed effects only 24 h after exposure, before any short-term effects from the initial exposure could resolve, so it is not possible to determine if the observed lung inflammation was transitory or persistent. More recent publications (e.g., Ilves et al. 2018 [16] and Park et al. 2018 [23]) evaluated longer-term responses, at 14- and 28-days post exposure. These studies demonstrate that for short-term CNF exposures, the initial inflammatory effects may subside by 28 days (similar to other PSLT dusts).

### 6.4. Lack of Dose–Response

For risk assessment purposes, a fundamental principle in the design of studies is to create a dose–response curve: Testing several concentrations of a material, including concentrations low enough that no effects are observed, all the way to high concentrations where adverse effects may be anticipated. Demonstrating a dose–response relationship associates the observed effects with material exposure, indicates at what concentrations a material might cause an effect such as lung inflammation, and allows evaluation of whether effects are likely to occur at realistic exposure levels. The in vivo inhalation studies were generally not performed according to international standard protocols. Standardized inhalation studies such as OECD 403 (acute), 412 (28-day) and 413 (90-day) typically require that at least three concentration levels be tested to allow for robust statistical analysis. Many of the animal studies published to date on CN dust inhalation tested just one or two doses and were not designed to evaluate and demonstrate a dose–response relationship, limiting their utility for risk assessment.

### 6.5. Control Groups

All dusts have the potential to irritate the lung when inhaled; therefore, a key question is whether CNs behave any differently or with greater potency compared to PSLT dusts or known inflammatory agents. This is assessed by including negative or positive control groups under similar conditions. Several of the reviewed studies of CNs are not relevant to the question because they did not include a conventional material, such as conventional cellulose, as a control. Ilves et al. (2018) was the first animal study to include reference material controls and demonstrated that, for short-term exposures at least, lung responses to CNF were similar to conventional cellulose in the study [16]. Studies that included positive control groups exposed to asbestos and MWCNT, demonstrated that CNs do not behave like these fibers, which caused a more potent and more persistent inflammatory response.

## 7. What the Studies Tell Us about the Risks of Inhaling Cellulose Nanomaterial in Dust in the Workplace 

There have been some advances in knowledge of the effects of short-term inhalation exposures of CN. The findings from Ilves et al. (2018) and Park et al. (2018) suggest that exposure to CNs result in transient inflammation similar to that caused by other PSLT dusts, such as cellulose, after short-term exposures; these effects are markedly different from those caused by fibers with known toxicity such as certain types of MWCNT or asbestos described by the fiber paradigm [16,23]. As summarized in Park et al. (2018), “Asbestos was the only material not properly handled by the immune system. Nanocellulose agents did not, as evidenced here, behave as “asbestos-like” materials” [23]. However, the studies conducted to date are limited in terms of their utility for risk assessment purposes because of their designs. Some uncertainty remains regarding: (i) Whether there are differential effects from exposure to different forms of CN (e.g., CNC versus CNF; or different surface functionalizations), (ii) the effects from low-dose, long-term CN inhalation exposure that more accurately reflects workplace conditions; and (iii) whether occupational exposure limits for conventional cellulose and other PSLT apply to CNs or should be modified. The available studies provide only a limited weight of evidence for risk assessment purposes but study quality is improving with time.

### 7.1. Cellular (In Vitro) Assays

Cellular studies allow researchers to investigate biological mechanisms in a highly controlled system, or to make comparisons between materials. To date, six studies (Table 1), have examined the effects of CN exposure on lung cells. These studies use cell lines derived from the lining of the lung, or immune cells normally found in lung tissue, to evaluate the effects of CN exposure, including endpoints such as viability and cytokine secretion. These models give an indication of whether the immune system might be triggered upon CN exposure; however, they do not capture the true complexity of an immune response in animals or people such as inflammation. In cellular studies with CN conducted up to 2017, the results varied and were often contradictory, with some studies indicating cellular toxicity and the potential for inflammation, and others not observing these effects. These discrepancies could be due to the cell culture model system [17], different exposure times and doses, exposure methods, purity, surface chemistry, and so forth. This limits the conclusions that can be drawn for risk assessment and highlights the importance of carefully designing and reporting study details.

In vitro systems are useful in providing supporting evidence for risk assessment purposes, including information on mechanisms of toxicity, and relative responses that allow grouping, screening, and prioritization [44,45]. Currently, animal models provide a more thorough understanding of how inhalation of CNs may interact with complex biological systems such as the lung; although, alternative testing strategies (non-animal tests) are rapidly being developed and validated to support risk assessment and reduce the number of animal studies [45].

### 7.2. Short-Term Animal Studies

Five of the seven animal studies reviewed evaluated exposure to CNC, and three of the studies evaluated CNF. All studies examined short-term exposures to CN and reported on potential inflammation in the lung by examining various tissue, cellular, and molecular endpoints. Other reported effects include reproductive effects [12], sex differences [13], and genotoxicity [14]. However, as outlined above, the conclusions drawn from these studies for risk assessment purposes are limited.

A key finding from the Ilves et al. (2018) and Park et al. (2018) studies, which also tested MWCNT and asbestos fibers, is that there is no strong evidence CNF and CNC belong to the harmful class of fibers described by the fiber paradigm [16,23]. The fiber paradigm is a structure:toxicity model which outlines specific physical and chemical properties of fibers associated with harmful effects (e.g., inflammation, fibrosis, and increased tumor incidence) when breathed into the lung; the effects occur when macrophages fail to phagocytize or engulf fibers, an event known as frustrated phagocytosis. In particular, the fiber paradigm applies to fibers thin enough to be respirable into the lung (<3 μm); long (>5 μm), rigid enough that they cause frustrated phagocytosis and cannot be cleared from the lung, and persistent enough that they remain in the lung [46,47]. While CNC and CNF do have typical widths <3 µm, CNCs are much shorter, generally on the scale of 100 s of nm in length, and, therefore, are not likely to result in frustrated phagocytosis [17]. CNFs are typically >1 µm in length but are not rigid and generally exist as complex, tangled networks of fibers [4,48]. Studies have found evidence that conventional cellulose, CNC and CNF are biopersistent in the lung [16,49,50]. However, in Ilves et al. (2018), in vivo exposure to CNF was not associated with a persistent inflammatory response, as is common with materials defined by the fiber paradigm [16]. The authors hypothesize that the gel-like state that CNF forms in water or the formation of a protein corona might render CNF biocompatible in the lung. Thus, although CNC and CNF widths are generally below 100 nm, other physical properties suggest that these materials do not likely adhere to the paradigm, much like other organic dusts [31,51]. Further measurements of rigidity, persistence, and inflammatory response over longer periods of time are needed to validate this hypothesis for different forms of CNs.

Short-term pulmonary exposures to CNC and CNF at high levels do result in an initial inflammatory response; although, the immune response is less strong and markedly different from that of asbestos and carbon nanotubes [16,23]. The immune response to CNF is substantially reduced after 28 days and is similar in response and duration to conventional cellulose exposure [16]. This suggests CNFs induce short-term inflammatory effects when breathed into the lung, similar to other PSLT dusts, including cellulose, which subside over time. A longer term in vivo study comparing CNC exposure to conventional cellulose has not yet been reported.

These are important findings that contribute to our understanding of the potential risks from inhalation of dried CNs in occupational environments. The results suggest that CNF causes an acute inflammatory reaction in the lung that resolves, similar to other PSLT dusts such as cellulose after short-term exposure. Further, both CNC and CNF elicit reactions in the lung after short-term exposures that are markedly different from fibers with known toxicity such as certain MWCNT or asbestos. However, there is still some uncertainty about the effects of breathing CN in dust and the mode of action, especially over the long term. Surface chemistry may be an important factor to consider, and CNF may be biopersistent—how this may affect hazard over the long term remains to be determined. In particular, the lack of long-term studies at realistic exposure levels and via inhalation rather than as a bolus dose leaves significant uncertainty about health effects despite the growing database.

## 8. Future Research and Recommendations 

Recent publications are improving the knowledge base, due to improved study designs that further our knowledge about the risks associated with inhaling CNs. These data suggest that CNF behaves similarly to other PSLT dusts such as cellulose for short-term exposures in the lung and that both CNC and CNF are markedly different from fibers with known toxicity, such as certain MWCNT, that meet the fiber paradigm. However, as outlined above, some data gaps still exist. Due to inadequate physical-chemical characterization, unrepresentative exposure methods, high dose and short duration testing, a lack of dose–response analysis, and the general absence of control groups, there is still uncertainty that limits the conclusions that can be drawn on the safety of CN in dry form in the workplace. For these reasons, the safest course of action is still to take a precautionary approach and prevent or minimize the potential for inhalation exposure. This includes adopting measures that are traditionally employed to avoid or mitigate workplace exposures to poorly soluble or insoluble dusts such as working with solutions and gel forms of CN when possible or isolating work with dusts to avoid breathing them. When not possible, measures such as enclosing mixing processes or wearing suitable personal protective equipment may be appropriate. The current data gaps and limitations for risk assessment are not specific to cellulose nanomaterials; these recommendations are applicable to most nanomaterials. Several organizations have published guidance on approaches to safely working with nanomaterials, including NIOSH in the United States and the World Health Organization.

Additional tools and studies needed to allow assessment of the potential health risks from occupational inhalation exposure include:

1. Better techniques to detect CNs in different media (e.g., air, water, biological fluids). It is challenging to measure occupational exposure to CNs due to their composition as organic carbohydrate materials. It can be difficult to identify and measure CNs, often present at very low levels, and to distinguish them from background sources of particles in the atmosphere. Detection and characterization of CN in the lung is required to understand biopersistence and clearance kinetics as they relate to any persistent negative responses, such as chronic inflammation.
2. More exposure assessments in industrial facilities. Several (unpublished) investigations indicate exposure levels are low in CN production environments. While techniques are not necessarily representative of industrial environments, studies by NIOSH in pilot CN facilities have not measured high levels of particles, and other studies have shown that common engineering control equipment such as fume hoods can effectively remove a large proportion of airborne CN. In one instance, particle counts were lower during the production of paper with CNF when compared to the control paper made without it [52]. Measurement of ambient levels of CNs typical of occupational exposures is critical to establishing appropriate dosing for long-term inhalation studies.
3. A long-term inhalation study mimicking realistic exposures. This includes using realistic exposure models (inhalation experiments instead of pharyngeal aspiration) that examine a range of realistic doses (show a dose–response) for different lengths of time, including a timepoint long enough to assess whether there is impairment when exposure occurs over time and whether recovery occurs, to distinguish transient from persistent effects. Control materials need to be included that compare CNs to conventional forms of cellulose to determine if there are any unique hazards associated with CNs or if they behave similarly and at a similar potency to other PSLT dusts. The use of positive and negative controls will also allow comparisons across materials and studies. These types of studies can be expensive but are needed to assess differences between CNs and conventional cellulose and other PSLT dusts.

## Figures and Tables

**Table 1:** Published studies examining effects of cellulose nanomaterial (CN) inhalation.

In Vivo Studies			In Vivo Studies		
First Author	Year	Material	First Author	Year	Material
Ilves	2018 [16]	cellulose nanofibrils (CNF)	Ilves	2018 [16]	CNF
Park	2018 [23]	CNF, cellulose nanocrystals (CNC)	Menas	2017 [20]	CNC, CNF
Catalan	2017 [15]	CNF	Lopes	2017 [21]	CNF
Shvedova	2016 [14]	CNC	Yanamala	2016 [19]	CNC, CNF
Farcas	2016 [13]	CNC	Endes	2014 [18]	CNC
Yanamala	2014 [12]	CNC	Clift	2011 [17]	CNC
O’Connor	2014 [22]	CNC			

**Table 2:** Summary of Krug and Wick study evaluation.

**Nanomaterial Characterization Score**	**Criteria**	**In Vivo Studies**									**In Vitro Studies**							
		**O’Connor 2014**	**Yanamala et al. 2014**	**Farcas et al. 2016**	**Shvedova et al. 2016**	**Catalan et al. 2017**	**Park et al. 2018**	**Ilves et al. 2018**	**TOTAL**	**%**	**Clift et al. 2011**	**Endes et al. 2014**	**Yanamala et al. 2016**	**Menas et al. 2017**	**Lopes et al. 2017**	**Ilves et al. 2018**	**TOTAL**	**%**
	Chemical composition, purity, impurities	0	1	1	2	0	1	2	7	50%	0	1	1	1	1	2	6	50%
	Particle size and size distribution	2	2	2	2	2	2	2	14	100%	2	2	2	2	2	2	12	100%
	Specific surface	2	0	0	0	0	0	0	2	14%	0	0	0	0	0	0	0	0%
	Morphology (crystalline/amorphous, shape)	2	2	0	2	2	2	2	12	86%	2	2	0	2	2	2	10	83%
	Surface chemistry, coating, functionalization	1	0	0	1	2	0	1	5	36%	0	1	0	0	2	1	4	33%
	Degree of agglomeration/aggregation and particle size distribution under experimental conditions (for example, media with/without proteins)	0	0	0	0	0	0	0	0	0%	0	1	0	0	2	0	3	25%
	Surface reactivity and/or surface load (zeta potential)	0	0	0	2	2	0	2	6	43%	0	0	0	0	2	2	4	33%
	Characterization Score (out of 14)	7	5	3	9	8	5	9		47%	4	7	3	5	11	9		46%
**Study Design Score**	**Criteria**	**In vivo studies**									**In vitro studies**							
		**O’Connor 2014**	**Yanamala et al. 2014**	**Farcas et al. 2016**	**Shvedova et al. 2016**	**Catalan et al. 2017**	**Park et al. 2018**	**Ilves et al. 2018**	**TOTAL**	**%**	**Clift *et al.* 2011**	**Endes et al. 2014**	**Yanamala et al. 2016**	**Menas et al. 2017**	**Lopes et al. 2017**	**Ilves et al. 2018**	**TOTAL**	**%**
	Applied concentration/dose, to be given in more than one unit.	0	0	0	0	0	0	2	2	17%	0	2	2	2	0	2	8	67%
	Doses should be clearly marked as “overload” or “non-overload”.	0	0	0	0	0	1	0	1	8%	NA	NA	NA	NA	NA	NA		
	At least two different tests for each biological end point.	1	1	1	1	2	1	1	8	67%	0	0	1	1	1	1	4	33%
	Study should contain data on the dose–effect relationship of the acute toxic effects.	0	0	0	0	1	0	0	1	8%	1	1	0	1	1	1	5	42%
	Interference of the nanomaterials with the test system.	0	0	0	0	0	0	1	1	8%	2	0	0	0	0	1	3	25%
	Evaluation that contaminants or solvents not responsible for observed toxicity	0	0	0	0	1	0	1	2	17%	0	2	1	1	1	2	7	58%
	Are doses relevant to human exposures?	0	0	0	0	0	1	0	1	8%	0	0	0	1	0	0	1	8%
	Positive Control	0	2	0	0	2	2	2	8	67%	2	2	2	2	2	2	12	100%
	Negative Control	0	0	0	0	2	0	2	4	33%	2	0	0	1	2	2	7	58%
	Vehicle Control	0	0	0	0	2	2	2	6	50%	2	2	0	0	0	2	6	50%
	Study Design Score (out of 20/18)	1	3	1	1	10	7	11		28%	9	9	6	9	7	13		49%

**Table 3:** Summary of Card and Magnuson nanomaterial score evaluation.

**Nanomaterial Characterization Score**	**Criteria**	**In Vivo Studies**							**In Vitro Studies**						**Total**	**%**
		**O’Connor 2014**	**Yanamala 2014**	**Farcas 2016**	**Shvedova 2016**	**Catalán 2017**	**Park 2018**	**Ilves 2018**	**Clift 2011**	**Endes 2014**	**Yanamala 2016**	**Menas 2017**	**Lopes 2017**	**Ilves 2018**		
	1. Agglomeration and/or aggregation	0	0	0	0	0	0	0	0	1	0	0	1	0	2	15%
	2. Chemical composition	0	0	0	1	0	0	1	0	1	0	0	0	1	4	31%
	3. Crystal structure/crystallinity	0	0	0	0	0	0	0	0	0	0	0	1	0	1	8%
	4. Particle size/size distribution	1	1	1	1	1	1	1	1	1	1	1	1	1	13	100%
	5. Purity	0	0	0	1	0	0	1	0	1	0	0	0	1	4	31%
	6. Shape	1	1	0	1	1	1	1	1	1	1	1	1	1	12	92%
	7. Surface area	1	0	0	0	0	0	0	0	0	0	0	0	0	1	8%
	8. Surface charge	0	0	0	1	1	0	1	0	1	0	0	1	1	6	46%
	9. Surface chemistry (including composition and reactivity)	0	0	0	0	0	0	0	0	0	0	0	1	0	1	8%
	10. Characterization completed in relevant experimental media	0	0	0	0	0	0	0	0	1	0	0	1	0	2	15%
	**Total**	**3**	**2**	**1**	**5**	**3**	**2**	**5**	**2**	**7**	**2**	**2**	**7**	**5**		**35%**
